# Supplementary material for: Pathogenicity of Different Betanodavirus RGNNV/SJNNV Reassortant Strains in European Sea Bass
Source: Pathogens. 2022 Apr 11;11(4):458. doi: 10.3390/pathogens11040458 (PMC9026442; doi:10.3390/pathogens11040458)
Supplement: Supplementary file 1 [file pathogens-11-00458-s001.zip › Table S1.pdf]

Table S1: Pairwise nucleotide distances estimated among sequences of the betanodavirus strains.

A) Pairwise nucleotide distances estimated among the RNA1 sequences of the betanodaviral strains used in the present study.

	283	132	292-7.8	292-1.2	187	367-2	61-48	188	461-1	165-6	540-7	484-2
**283**												
**132**	0.031											
**292-7.8**	0.035	0.005										
**292-1.2**	0.036	0.005	0.000									
**187**	0.039	0.011	0.008	0.008								
**367-2**	0.038	0.011	0.016	0.016	0.020							
**61-48**	0.038	0.007	0.007	0.007	0.012	0.019						
**188**	0.039	0.011	0.008	0.008	0.000	0.020	0.012					
**461-1**	0.035	0.007	0.005	0.005	0.010	0.014	0.008	0.010				
**165-6**	0.043	0.012	0.011	0.012	0.015	0.025	0.016	0.015	0.010			
**540-7**	0.185	0.180	0.186	0.186	0.186	0.185	0.184	0.186	0.180	0.187		
**484-2**	0.189	0.181	0.186	0.186	0.186	0.191	0.184	0.186	0.180	0.190	0.017	

B) Pairwise nucleotide distances estimated among the RNA2 sequences of the betanodaviral

	283	132	292-7.8	292-1.2	187	367-2	61-48	188	461-1	165-6	540-7	484-2
**283**												
**132**	0.215											
**292-7.8**	0.214	0.014										
**292-1.2**	0.215	0.015	0.001									
**187**	0.213	0.016	0.009	0.010								
**367-2**	0.213	0.011	0.012	0.012	0.015							
**61-48**	0.216	0.019	0.009	0.010	0.014	0.017						
**188**	0.213	0.016	0.009	0.010	0.000	0.015	0.014					
**461-1**	0.209	0.015	0.009	0.010	0.012	0.014	0.013	0.012				
**165-6**	0.214	0.018	0.010	0.011	0.014	0.017	0.015	0.014	0.014			
**540-7**	0.215	0.032	0.034	0.035	0.036	0.031	0.038	0.036	0.035	0.038		
**484-2**	0.220	0.033	0.036	0.036	0.038	0.042	0.039	0.038	0.041	0.039	0.010	

strains used in the present study.
