# Supplementary material for: Pathogenicity of Different Betanodavirus RGNNV/SJNNV Reassortant Strains in European Sea Bass
Source: Pathogens. 2022 Apr 11;11(4):458. doi: 10.3390/pathogens11040458 (PMC9026442; doi:10.3390/pathogens11040458)
Supplement: Supplementary file 1 [file pathogens-11-00458-s001.zip › Table S2.pdf]

Table S2: Pairwise amino acid distances estimated among sequences of the betanodavirus strains.

A) Pairwise amino acid distances estimated among the RNA1 sequences of the betanodaviral strains used in the present study.

	283	132	292-7.8	292-1.2	187	367-2	61-48	188	461-1	165-6	540-7	484-2
**283**												
**132**	0.016											
**292-7.8**	0.020	0.003										
**292-1.2**	0.021	0.003	0.001									
**187**	0.023	0.006	0.003	0.004								
**367-2**	0.023	0.005	0.013	0.013	0.016							
**61-48**	0.021	0.005	0.004	0.005	0.006	0.015						
**188**	0.023	0.006	0.003	0.004	0.000	0.016	0.007					
**461-1**	0.019	0.003	0.002	0.002	0.005	0.008	0.003	0.005				
**165-6**	0.028	0.008	0.008	0.009	0.007	0.021	0.012	0.007	0.007			
**540-7**	0.135	0.136	0.142	0.142	0.142	0.141	0.141	0.141	0.135	0.145		
**484-2**	0.146	0.134	0.142	0.142	0.142	0.150	0.141	0.141	0.133	0.148	0.012	

B) Pairwise amino acid distances estimated among the RNA2 sequences of the betanodaviral

	283	132	292-7.8	292-1.2	187	367-2	61-48	188	461-1	165-6	540-7	484-2
**283**												
**132**	0.263											
**292-7.8**	0.268	0.011										
**292-1.2**	0.268	0.011	0.000									
**187**	0.265	0.011	0.009	0.009								
**367-2**	0.275	0.011	0.018	0.018	0.018							
**61-48**	0.259	0.009	0.007	0.007	0.007	0.016						
**188**	0.265	0.011	0.009	0.009	0.000	0.018	0.007					
**461-1**	0.256	0.009	0.007	0.007	0.007	0.016	0.000	0.007				
**165-6**	0.265	0.011	0.009	0.009	0.013	0.018	0.011	0.013	0.011			
**540-7**	0.268	0.032	0.039	0.039	0.039	0.034	0.036	0.039	0.036	0.039		
**484-2**	0.278	0.030	0.037	0.037	0.037	0.043	0.034	0.037	0.041	0.037	0.011	

strains used in the present study.
